# Incidence of Type 1 Diabetes among Children and Adolescents in Italy between 2009 and 2013: The Role of a Regional Childhood Diabetes Registry

**DOI:** 10.1155/2016/7239692

**Published:** 2016-03-22

**Authors:** F. Fortunato, M. G. Cappelli, M. M. Vece, G. Caputi, M. Delvecchio, R. Prato, D. Martinelli, Apulian Childhood-Onset Diabetes Registry Workgroup

**Affiliations:** ^1^Department of Medical and Surgical Sciences, University of Foggia, Viale Pinto 1, 70121 Foggia, Italy; ^2^Taranto Local Health Unit, Viale Virgilio 31, Taranto, 74121 Puglia, Italy; ^3^Pediatric Department “B. Trambusti”, Policlinico Hospital, Piazza Giulio Cesare 11, 70124 Bari, Italy; ^4^Pediatric Department, “Vito Fazzi” Hospital, Piazzetta Muratore, 73100 Lecce, Italy; ^5^Pediatric Department, “F. Ferrari” Hospital, Via F. Ferrari 1, Casarano, 73042 Lecce, Italy; ^6^Department of Biomedical Sciences and Human Oncology, Policlinico Hospital, Giovanni XXIII Children's Hospital, Via Amendola 207, 70126 Bari, Italy; ^7^Pediatric Department, “Ospedali Riuniti” Policlinico Hospital, Viale Pinto 1, 70122 Foggia, Italy; ^8^Pediatric Department, “Dario Camberlingo”Hospital, Viale M. delle Grazie, Francavilla Fontana, 72021 Brindisi, Italy; ^9^Pediatric Department, “T. Maselli” Hospital, Viale 2 Giugno, San Severo, 71016 Foggia, Italy; ^10^Pediatric Department, “Di Summa-Perrino” Hospital, S.S. 7 per Mesagne, 72100 Brindisi, Italy; ^11^Pediatric Department, “G. Panico” Hospital, Via S. Pio X 4, Tricase, 73039 Lecce, Italy; ^12^Department of Metabolic Diseases, Clinical Genetics and Diabetology, Policlinico Hospital, Giovanni XXIII Children's Hospital, Via Amendola 207, 70126 Bari, Italy

## Abstract

*Background.* Surveillance represents a key strategy to control type 1 diabetes mellitus (T1DM). In Italy, national data are missing. This study aimed at evaluating the incidence of T1DM in subjects <18 year olds in Apulia (a large southeastern region, about 4,000,000 inhabitants) and assessing the sensitivity of the regional Registry of Childhood-Onset Diabetes (RCOD) in the 2009–2013 period.* Methods.* We performed a retrospective study matching records from regional Hospital Discharge Registry (HDR), User Fee Exempt Registry (UFER), and Drugs Prescription Registry (DPR) and calculated T1DM incidence; completeness of each data source was also estimated. In order to assess the RCOD sensitivity we compared cases from the registry to those extracted from HDR-UFER-DPR matching.* Results.* During 2009–2013, a total of 917 cases (about 184/year) in at least one of the three sources and an annual incidence of 25.2 per 100,000 were recorded, lower in infant, increasing with age and peaked in 5- to 9-year-olds. The completeness of DPR was 78.7%, higher than that of UFER (64.3%) and of HDR (59.6%). The RCOD's sensitivity was 39.05% (360/922; 95% CI: 34.01%–44.09%).* Conclusions.* Apulia appeared as a high-incidence region. A full, active involvement of physicians working in paediatric diabetes clinics would be desirable to improve the RCOD performance.

## 1. Introduction

Type 1 diabetes mellitus (T1DM), previously known as insulin-dependent, is a chronic disease that usually develops during childhood and adolescence. The disease is characterized by a deficit of insulin production and requires lifelong administration of insulin injections for survival [1, 2]. Uncontrolled diabetes can seriously damage many of the body's systems, especially the nerves and blood vessels, leading over time to severe chronic conditions and early death with a large social and economic impact [1].

As one of the major chronic diseases during the age of development, with about 350 million people affected worldwide [1], the diabetes mellitus represents a public health problem in both low- and high-income countries [3]. The incidence varies significantly among countries, and even among regions within countries [4, 5]. During the first half of the 1990s, the overall age-adjusted incidence rate of T1DM varied globally from 0.1 in China and Venezuela to 37 per 100,000/year in Finland [6]. In Finland, after a modest increase up until 1988, the incidence increased annually by 3.6% until 2005, followed by a plateau until the end of 2011 (average incidence 2006–2011: 62.5 per 100,000 person-years, 68.4 per 100,000 person-years among boys, and 55.4 per 100,000 person-years among girls) [7]. A 2008 study conducted in 19 European countries showed that the incidence among children aged <15 years in the 2004–2008 period varied from 5.8 per 100,000/year in Macedonia to 36.6 per 100,000/year in Sweden [8]. More recent estimations assessed that in the United States in 2012 0.25% of people aged <20 years were diagnosed with diabetes [9]. In Norway, in the period 2004–2012 the average incidence rate of T1DM in children below 15 years of age was 32.7 per 100,000 person-years [10].

In Italy, although several authors have reported T1DM incidence data from selected geographical areas since the 1980s, national incidence rates are still missing [11]. In their study, Vichi et al. estimated a mean nationwide incidence rate of 13.4 among Italian children aged 0–4 years in the period 2005–2010, using the first hospital admission for T1DM as a proxy of a new T1DM case [11]. In the Veneto region, identified as an area with intermediate-high risk for T1DM, an incidence rate of 16.5 per 100,000 person-years was reported among children aged 0–18 years in the 2008–2012 period [12]. In Sardinia, the highest-incidence Italian region, the average yearly standardized incidence rate of T1DM is 38.8/100,000 [9–22].

Surveillance of diabetes represents one of the key strategies to control the disease [1]. The systematic collection of diabetes mellitus cases provides a good instrument to define the spatial and temporal trends of the disease and to assess the needs in terms of health care intervention. Estimating the burden of T1DM by compiling a registry is an opportunity because it is particularly suitable for being captured. The disease is neither too severe nor too frequent; it also has a classic set of symptoms, and it can be rapidly diagnosed by simple tests. Basic information needed for registration is name, date of birth, date of diagnosis, place of residence, and ascertainment status [14].

In 1997, a Registry of Childhood Type 1 Diabetes Mellitus in Italy (RIDI) was established to coordinate preexisting registries and to promote the setting up of new local registries [15]. Until now, RIDI has included a total of seven regional registries (Liguria, Marche, Umbria, Lazio, Abruzzo, Campania, and Sardinia) and five provincial registries (Trento, Turin, Pavia, Modena, and Florence-Prato) [15]. In Apulia, a southeastern Italian region of about 4,000,000 inhabitants, new cases of T1DM in patients aged 0–17 years have been recorded in the Registry of Childhood-Onset Diabetes (RCOD) since 2009.

This study aimed at estimating the incidence of T1DM with onset before 18 years of age in Apulia region by using routinely available epidemiological data sources and assessing the sensitivity of the Regional Childhood-Onset Diabetes Registry in the period 2009–2013.

## 2. Methods

### 2.1. Estimate of the Incidence of T1DM

#### 2.1.1. Data Sources

In order to estimate the incidence of T1DM we performed a retrospective study by using three data sources:

- *Hospital Discharge Registry* (HDR), which collects data on discharge diagnoses (one main and up to five secondary diagnoses) and procedures of all patients admitted to hospitals: we extracted records of patients aged <18 years resident in Apulia discharged with a diagnosis of T1DM (ICD9-CM codes 250.x1 and 250.x3) as either main or secondary diagnosis for the period 2004–2013.
- *User Fee Exempt Registry* (UFER), in which information on chronic patients entitled to fee exemption for medical consultations and drugs for their specific medical condition was collected: each condition was identified by a specific and unique code. We extracted records of subjects aged <18 years resident in Apulia entitled to fee exemption for diabetes (UFER code: 013) in 2013, regardless of the date of the first diagnosis.
- *Drugs Prescription Registry* (DPR), where information on drugs prescribed to patients by the health services was recorded: drugs were coded using the Anatomical Therapeutic Chemical Classification (ATC). We extracted records of subjects aged <18 years resident in Apulia with a presumed first drugs prescription for insulin or analogues (ATC code: A10A) in the period 2004–2013.

#### 2.1.2. Procedure

We calculated the annual standardized hospitalization rates (number of hospitalizations/number of residents per 1,000 Italian populations) in the period 2009–2013. The mid-year estimates of Apulian and Italian populations were obtained from ISTAT (Italy's National Census Bureau) estimate.

**Figure 1:**
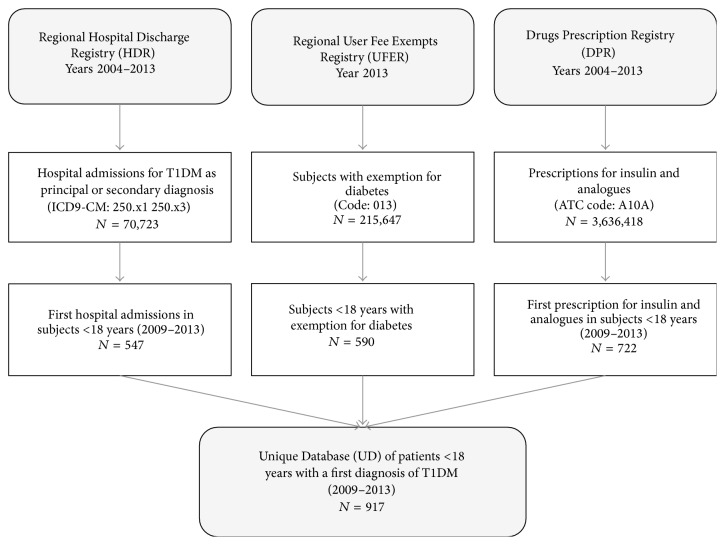
Flowchart of the creation of the total number of patients <18 years with T1DM (the Unique Database). Apulia, Italy, 2009–2013.

In order to estimate T1DM incidence in the period 2009–2013, we created a Unique Database (UD) matching the records extracted from the three data sources by using personal ID number as linkage key (Figure 1). In order to ensure that only new T1DM diagnoses were extracted, we performed a retrospective data cleansing by comparing data from the period 2009–2013 with that from 2004–2008; we identified duplicates by using the personal ID number as linkage key.

#### 2.1.3. Statistical Analysis

Annual crude and specific, by sex and group of age, incidence rates were calculated by dividing the UD cases by the number of residents in Apulia for the period 2009–2013. In order to assess the effects of age, gender, and calendar year, a Poisson regression model was performed by using STATA SE 14.1, considering *p* values of <0.05 as significant.

Moreover, the completeness of each source (sensitivity) was estimated by dividing the number of T1DM cases observed in each source by the total number of patients in the UD.

### 2.2. Sensitivity of the Apulian RCOD

The RCOD is currently fed by a network of 13 paediatricians working in nine out of the 30 regional paediatric departments and one endocrinologist who works in one of the seven departments of endocrinology.

A case of diabetes mellitus was defined using the following criteria:

- Symptoms of marked hyperglycaemia including polyuria, polydipsia, weight loss, sometimes with polyphagia, and blurred vision [16, 23].
- A1C ≥ 6.5% or Fasting Plasma Glucose (FPG) ≥ 126 mg/dL (7.0 mmol/L) where fasting is defined as no caloric intake for at least 8 h or 2 h plasma glucose ≥ 200 mg/dL (11.1 mmol/L) during an Oral Glucose Tolerance Test (OGTT): the test was performed as described by the WHO, using a glucose load containing the equivalent of 75 g anhydrous glucose dissolved in water or in a patient with classic symptoms of hyperglycaemia or hyperglycaemic crisis, a random plasma glucose ≥200 mg/dL (11.1 mmol/L) [15, 23].
- Insulin dependence and positivity for autoantibodies that are common in T1DM:
  - Islet Cell Antibody (ICA),
  - Glutamic Acid Decarboxylase (GAD65) autoantibody,
  - Insulin Autoantibody (IAA),
  - Islet Antigen 2 (IA-2) autoantibody [17],
  - Zinc Transporter 8 (ZnT8) autoantibody [24].

Data are recorded by using an online data entry platform available by password authentication on the institutional website of the Regional Observatory for Epidemiology.

The main information collected in the RCOD is physician contact details, demographic characteristics of the patient (including personal ID number), date of first diagnosis, department and hospital of diagnosis, values of pH, positivity for ICA, GAD65 autoantibody, IAA, IA-2 autoantibody, ZnT8 autoantibody, comorbidities, family history, and date of record creation.

In order to assess the sensitivity of the RCOD, we extracted all cases of T1DM registered in the period 2009–2013 and we matched them with the UD by using the personal ID number as linkage key. We also assessed the level of completeness of each variable collected in the RCOD and the timeliness of registration by calculating the average time between the date of first diagnosis and the date of record creation.

### 2.3. Ethics

The study was approved by the Institutional Review Board of the Apulian Observatory for Epidemiology. It was conducted in accordance with the Guidelines for Good Clinical Practice and the ethical principles originating in the Declaration of Helsinki.

## 3. Results

### 3.1. Estimate of the Incidence of T1DM

Between 2009 and 2013, in Apulia, we identified a total of 4,642 hospitalizations for diabetes mellitus in subjects aged <18 years, of which 4,255 for T1DM, with an average of 851 admissions/year and an annual standardized hospitalization rate of 1.2 per 1,000. The average number of admissions per patient was 6 (range: 1–32). After cleansing of duplicates, we identified 547 patients who had been discharged with a primary diagnosis of probable T1DM.

A total of 590 subjects <18 years old entitled to fee exemption for diabetes were recorded in 2013.

Drugs prescriptions for insulin or analogues recorded were 40,195 (annual average: 8,039), accounting for a total of 722 (annual average: 144) subjects aged <18 years with a presumed first prescription between 2009 and 2013.

In the study period, a total of 917 cases (about 184/year) were recorded in at least one of the three sources. The estimated average annual incidence rate was 25.2 per 100,000 (25.7 per 100,000 males and 24.4 per 100,000 females, resp.) and progressively decreased in the study period (*p* < 0.05; Tables 1 and 2). It was lower in children aged <1 year, increased with age, and peaked in children aged from 5 to 9 years (*p* < 0.05; Tables 1 and 2).

**Figure 2:**
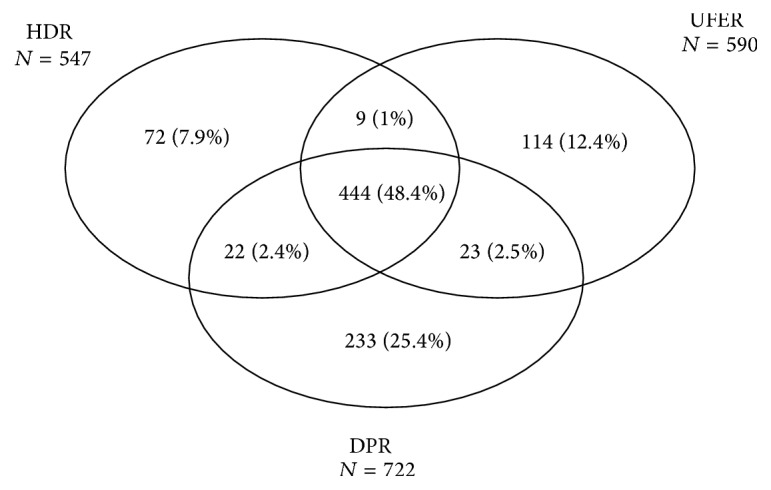
Contribution of HDR, UFER, and DPR to the creation of the total number of patients <18 years with T1DM (the Unique Database). Apulia, Italy, 2009–2013.

The contribution of each data source to the UD is showed in Figure 2. 48.4% of patients were identified in all three sources, 5.9% in two of the three sources (HDR/UFER or HDR/DPR or DPR/UFER), and 45.7% in one source (HDR or UFER or DPR). The sensitivity of DPR was 78.7%, higher than that of UFER (64.3%) and of HDR (59.6%).

### 3.2. Sensitivity of the Apulian RCOD

During 2009–2013, 360 new cases of T1DM were recorded in the RCOD (50.8% males) with an average age at diagnosis of 8.6 years (SD = ±4.1; range 0–17 years). Most cases were aged between 5 and 9 years (38.6%; *N* = 139) and 10–14 years (34.2%; *N* = 123), 18.3% (*N* = 66) were 1–4 years old, and 8.3% (*N* = 30) were 15–17 years old; only two cases were <2 years (0.5%).

11.1% (*N* = 40) of patients' relatives had a diagnosis for T1DM, 13.9% (*N* = 50) for T2DM, 7.5% (*N* = 27) for thyroid disease, 1.7% (*N* = 6) for rheumatoid arthritis, and 3.6% (*N* = 13) for celiac disease. 8% (*N* = 29) of enrolled patients were also affected by celiac disease, 7.5% (*N* = 27) were affected by thyroiditis, 0.8% (*N* = 3) were obese, and two suffered from Arnold Chiari Syndrome Type I. 16.7% (*N* = 18/108) of cases tested positive for ICA, 69.8% (*N* = 164/235) for GAD65 autoantibody, 49.5% (*N* = 102/206) for IAA, 53.6% (*N* = 15/28) for IA-2 autoantibody, and 33.3% (*N* = 1/3) for ZnT8 autoantibody.

After record linkage between the RCOD and the UD, we identified a total of 922 patients aged <18 years. The average sensitivity of the RCOD was 39.05% (360/922; 95% CI: 34.01%–44.09%). The UD missed 5 cases. The RCOD missed a total of 562 patients (mean age = 10.3 ± 4.9 years, 52.8% males) of which 35.2% (198/562) were hospitalized, 43.8% (246/562) were entitled to fee exemption for diabetes, and 66.4% (373/562) had drugs prescriptions for insulin or analogues.

The level of completeness was >90% for all the RCOD variables with the exception of “pH,” available for 232/360 patients. The average delay was 16 months (range 0–57.2 months).

## 4. Discussion

With an estimated average annual incidence rate of 25.2 per 100,000 inhabitants, Apulia appeared as a high-incidence region. This is in contrast with previous studies reporting that the lowest T1DM rates in Italy were observed in the southern regions [15]. Lower incidence rates were recorded in Lombardy (7.2/100,000 per year) and Marche (9.7/100,000 per year) regions during the DiaMond project conducted from 1990 to 1994 in children <14 years of age [18] and the RIDI study (12.26 per 100,000 person-years in the period 1990–2003 among children 0–14 years old) [19]. Worldwide, the highest rates were found in Sardinia, Italy (38.8/100,000 per year) [9–22], and in some European Nordic countries (Finland, Sweden, and Norway) [18]. Whether differences in incidence of type 1 diabetes in Italy could be attributed to genetic differences and to an increase in the prevalence of susceptible genes, due to improved survival, or to a different distribution of nongenetic factors and environmental determinants of the disease, such as infections, nutritional components, and toxins, remains to be clarified [15, 25, 26].

Compared with other studies [11, 15], we found a higher, though not statistically significant, incidence rate in boys rather than in girls. Regarding the age effect, we found a high incidence in infants, in contrast with some previous studies where development of T1DM in the first years of life was considered very rare but in accordance with the global increase in the incidence in very young children [27].

In contrast to the trends recorded in most populations worldwide from 1989 to 2003 [6, 13], in Apulia the incidence rate seemed to reduce over time (from 36.6 in 2009 to 20.2 in 2013). However, these results should be interpreted with caution because the period covered in our analysis was too short to accumulate enough cases for appropriate evaluations.

In the period 2009–2013, in Apulia region, we selected from three sources (HDR, UFER, and DPR) 917 cases of T1DM, of which 355 were identified as new cases by matching with the RCOD. As shown in other studies, administrative health data are an efficient tool to assess epidemiologic trends in the population and a good source of population-based information for research about disease and for public health surveillance [20–22]. The combination of sources in our study was fairly original when compared to the other experiences cited in the literature [28–32]. An Italian study by Ballotari et al. [28] showed that several data sources made a meaningful contribution to assess the burden of diabetes (HDR, UFER, DPR, biochemistry lab, outpatient clinics, and mortality database), capturing cases not otherwise identified. This could explain why the incidence rate in children aged 0–4 years was higher in our analysis than in previous studies that estimated incidence by using only HDR (13.4 × 100,000 in Italy, 8.9 × 100,000 in Apulia [11]). According to our findings, the DPR sensitivity estimated by Ballotari et al. was more than 70% [28]. A study in 2014 by Rawshani et al. suggested that DPR could be considered the gold standard for monitoring the incidence of T1DM due to its feasibility, reliability, and cost-effectiveness [26]. As a matter of fact, all individuals with Type 1 diabetes should receive insulin, and it is quite impossible to do so in Italy without having been visited and having received a prescription by a paediatrician or a general practitioner.

However, administrative data was not originally intended to epidemiological purpose and there could be several limitations in their use for the evaluation of the incidence of the disease, including errors at each step of the coding process [33]. Another limitation of our study was related to the different probability of a case being included on each source, making it inappropriate to adopt the capture-recapture methodology as a means to monitor the diabetes epidemic [26, 34, 35]. In Apulia, the probability to be hospitalized is higher among severe cases than the others; it is not comparable to that of taking drugs (all individuals should receive insulin) or that of being entitled to a fee exemption [36].

In accordance with larger national and international experiences [10, 30, 37], the Apulian RCOD implemented in 2009 allowed the identification, based on a clinical diagnosis, of new cases of T1DM in patients aged <18 years, not present in the routine data sources. In our experience, only five cases were identified through RCOD, most probably not hospitalized and not entitled to fee exemption for chronic medical condition in 2013. As highlighted by a study of Hodgson et al. in the UK, despite the fact that HDR represents a useful instrument in exploring the epidemiology of diabetes, it is crucial to establish dedicated diabetes registries. A diabetes registry could incorporate additional data for undertaking etiological research into this important childhood condition [38]. Although the Apulian RCOD has shown a level of clinical documentation completeness >90%, a sensitivity of 39% is still low to ensure reliable epidemiological data, firstly because of the limited number of physicians involved in the activities.

Since the RCOD makes the collection of useful information for clinical management and follow-up of T1DM patients possible, the active involvement of all physicians working in Apulian paediatric diabetes clinics would be desirable. Periodical feedback of epidemiological reports from the RCOD might help increase physicians' awareness and participation in the network.

## Figures and Tables

**Table 1:** Estimated T1DM incidence rates (per 100,000) among subjects <18 years, by sex, year, and age group. Apulia, Italy, 2009–2013.

	Males			Females			All		
	*N*	Rate	95% CI	*N*	Rate	95% CI	*N*	Rate	95% CI
Year									
2009	141	36.4	30.4–42.4	135	36.9	30.6–43.1	276	36.6	32.3–40.9
2010	106	27.8	22.5–33.0	97	26.8	21.5–32.2	203	27.3	23.5–31.1
2011	98	26.1	20.9–31.2	77	21.8	16.9–26.7	175	24.0	20.4–27.6
2012	57	15.2	11.2–19.1	62	17.6	13.2–21.9	119	16.3	13.4–19.3
2013	82	22.4	17.5–27.2	62	18.0	13.5–22.5	144	20.2	16.9–23.6
Age group									
<1 year	8	8.8	2.7–14.8	9	10.4	3.6–17.2	17	9.6	5.0–14.1
1–4 years	77	20.2	15.7–24.7	72	19.9	15.3–24.5	149	20.1	16.8–23.3
5–9 years	148	29.0	24.3–33.7	148	30.4	25.5–35.3	296	29.7	26.3–33.1
10–14 years	162	29.8	25.2–34.4	137	26.6	22.2–31.1	299	28.2	25.0–31.4
15–17 years	89	26.0	20.6–31.4	67	20.8	15.8–25.7	156	23.5	19.8–27.1

**Table 2:** Incidence Rate Ratio (IRR) with 95% CI of T1DM among subjects <18 years, by sex, year, and group of age. Apulia, Italy, 2009–2013.

	IRR	*z*	*p*	95% CI
Sex				
Female	Ref.			
Male	1.06	0.88	0.381	0.93–1.21
Year				
2009	Ref.			
2010	0.75	−3.18	0.001	0.62–0.89
2011	0.66	−4.34	0.000	0.54–0.79
2012	0.45	−7.25	0.000	0.36–0.56
2013	0.55	−5.87	0.000	0.45–0.67
Age group				
<1 year	Ref.			
1–4 years	2.11	2.91	0.004	1.28–3.48
5–9 years	3.12	4.56	0.000	1.91–5.08
10–14 years	2.98	4.37	0.000	1.83–4.85
15–17 years	2.46	3.52	0.000	1.49–4.05

## Appendix

### Apulian Childhood-Onset Diabetes Registry Workgroup

Benelli Marzia, Pediatric Department, “Vito Fazzi” Hospital, Piazzetta Muratore, 73100 Lecce, Italy. Cardinale Giuliana and Ponzi Giuseppe, Pediatric Department, “F. Ferrari” Hospital, Via F. Ferrari 1, 73042 Casarano, Lecce, Italy. Cavallo Luciano, Lonero Antonella and Zecchino Clara, Department of Biomedical Sciences and Human Oncology, Policlinico Hospital, Giovanni XXIII Children's Hospital, Via Amendola 207, 70126 Bari, Italy. Cioccia Tilde, Pediatric Department, “Ospedali Riuniti” Policlinico Hospital, Viale Pinto 1, 70122 Foggia, Italy. Coccioli Maria Susanna, Pediatric Department, “Dario Camberlingo”Hospital, Viale M. delle Grazie, 72021 Francavilla Fontana, Brindisi, Italy. Di Pumpo Raffaele and Mariano Matteo, Pediatric Department, “T. Maselli” Hospital, Viale 2 Giugno, 71016 San Severo, Foggia, Italy. Gallo Francesco, Pediatric Department, “Di Summa-Perrino” Hospital, S.S. 7 per Mesagne, 72100 Brindisi, Italy. Ingletto Dario, Pediatric Department, “G. Panico” Hospital, Via S. Pio X 4, 73039 Tricase, Lecce, Italy. Piccinno Elvira, Department of Metabolic Diseases, Clinical Genetics and Diabetology, Policlinico Hospital, Giovanni XXIII Children's Hospital, Via Amendola 207, 70126 Bari, Italy.
